# Miniaturized EBG Antenna for Efficient 5.8 GHz RF Energy Harvesting in Self-Powered IoT and Medical Sensors

**DOI:** 10.3390/s25154777

**Published:** 2025-08-03

**Authors:** Yahya Albaihani, Rizwan Akram, Abdullah. M. Almohaimeed, Ziyad M. Almohaimeed, Lukman O. Buhari, Mahmoud Shaban

**Affiliations:** Department of Electrical Engineering, College of Engineering, Qassim University, Buraydah 51452, Saudi Arabia; 421116562@qu.edu.sa (Y.A.); r.akram@qu.edu.sa (R.A.); z.mohaimeed@qu.edu.sa (Z.M.A.); 451116402@qu.edu.sa (L.O.B.); s.mahmoud@qu.edu.sa (M.S.)

**Keywords:** miniaturized antenna design, wireless power transfer (WPT), metamaterial-inspired antennas, electromagnetic band gap, 5.8 GHz ISM band, RF energy harvesting, internet of things, radiation efficiency, self-powered IoT sensor, battery-less devices

## Abstract

This study presents a compact and high-efficiency microstrip antenna integrated with a square electromagnetic band-gap (EBG) structure for radio frequency energy harvesting to power battery-less Internet of Things (IoT) sensors and medical devices in the 5.8 GHz Industrial, Scientific, and Medical (ISM) band. The proposed antenna features a compact design with reduced physical dimensions of 36 × 40 mm^2^ (0.69λ_o_ × 0.76λ_o_) while providing high-performance parameters such as a reflection coefficient of −27.9 dB, a voltage standing wave ratio (VSWR) of 1.08, a gain of 7.91 dBi, directivity of 8.1 dBi, a bandwidth of 188 MHz, and radiation efficiency of 95.5%. Incorporating EBG cells suppresses surface waves, enhances gain, and optimizes impedance matching through 50 Ω inset feeding. The simulated and measured results of the designed antenna show a high correlation. This study demonstrates a robust and promising solution for high-performance wireless systems requiring a compact size and energy-efficient operation.

## 1. Introduction

**Figure 1:**
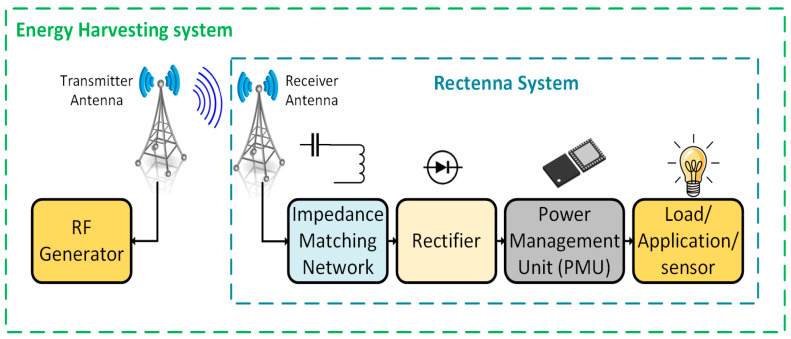
The primary components of WPT and RFEH systems.

The rapid development of 5G technology, the Internet of Things (IoT), the fourth industrial revolution, and smart city ecosystems has intensified the demand for autonomous, battery-free devices capable of operating in energy-constrained environments [1,2,3]. IoT networks have billions of sensors utilized for environmental monitoring, industrial automation, and healthcare, which require sustainable power solutions to overcome the limitations of conventional batteries, such as their finite lifespan, maintenance costs, and environmental hazards [4]. Wireless power transfer (WPT) and RF energy harvesting (RFEH) have emerged as transformative technologies, enabling devices to scavenge energy from ambient electromagnetic signals emitted by Wi-Fi routers, cellular towers, and IoT transceivers [5,6,7]. Figure 1 shows the main components of WPT and RFEH [8]. The antenna plays a crucial role in both systems, where the receiving antenna at the RFEH system captures ambient radio frequency signals from the environment for energy harvesting.

Among the available frequency bands from environment, the 5.8 GHz Industrial, Scientific, and Medical (ISM) band offers distinct advantages, including global regulatory compliance, reduced interference compared to the overcrowded 2.4 GHz band, lower latency, a high data rate, and moderate propagation loss [9], making it ideal for applications ranging from medical telemetry to indoor energy harvesting.

Among the numerous antennas available, such as dipole, monopole, Yagi–Uda, loop, meander, and horn antennas, the Microstrip Patch Antenna (MPA) type has been chosen for this study due to its unique features and advantages. MPAs are a type of planar antenna widely used in wireless applications. They are favored for their low profile, lightweight design, and ease of fabrication, making them suitable for various modern technologies. However, they face limitations such as the narrow bandwidth, low gain, and surface wave propagation [10]. Numerous research studies have been reported in the literature to address these unwanted characteristics. Despite the utilization of low-dielectric substrates in the microstrip antenna designs, the surface wave excitation of these antennas reduces their efficiency and gain. In contrast, a high dielectric substrate can be used in the design, resulting in a limited bandwidth and undesired surface wave excitation [11]. In addition, balancing miniaturization with performance parameters such as gain, directivity, bandwidth, and efficiency remains a challenge, particularly for medical devices and conformal IoT sensors [4,12].

To address these challenges, recent advancements have focused on meta-materials such as split-ring resonators (SRRs) [13,14,15] and complementary split-ring resonators (CSRRs) [16,17,18], Left-Handed Metamaterials [19,20], Metasurfaces [21], and electromagnetic band-gap (EBG) structures, which can be integrated into conventional antenna designs to manipulate electromagnetic waves in ways that natural materials cannot [11,22,23,24,25,26,27]. EBG structures have become of considerable interest in recent years, and various types have been validated. Generally, they can be classified into three categories based on their geometric configuration: 3D volumetric structures, 2D planar surfaces, and 1D transmission lines. Due to their low profile and ease of manufacture, two-dimensional surfaces are widely employed in the design of EBG structures and antenna design [25].

EBG structures are advanced periodic (or non-periodic) conductive patterns incorporated into antenna designs to enhance performance by suppressing surface waves and reducing substrate losses while improving radiation efficiency, which is critical for compact high-performance antennas in applications such as medical devices, ISM applications, and IoT sensors [28,29]. EBG structures prevent or allow electromagnetic (EM) wave propagation within a specific frequency range. The operation frequency of the antenna should lie in the band gap of the EBG structure to eliminate the surface waves. Consequently, the EBG structure’s design parameters must fall within specific ranges. Thus, the EBG structure and the antenna must be designed to match each other.

Several studies have focused on single-resonance frequency antennas at 5.8 GHz with EBG structures. For instance, the feasibility of utilizing an EBG-based MPA for gain enhancement was investigated by the authors of [30], where a 3 × 3 EBG array considerably improved the gain from 1.8 dBi to 11.4 dBi and aperture efficiency from 3.2% to 29.6% at 5.8 GHz. Similarly, others focused on dual-band antennas at 2.45 GHz and 5.8 GHz, incorporating EBG structures such as a small-sized embedded EBG structure for wearable applications in the ISM dual band, which was designed by the authors of [31], which effectively reduced the specific absorption rate (SAR) while maintaining efficient radiation characteristics. In contrast, a novel multi-band compact slot antenna with an EBG structure was proposed in [27], covering frequencies including 5.8 GHz for wearable applications. Moreover, wideband antennas at 5.8 GHz with EBG structures have also been developed. A defected ground EBG-based fractal patch antenna was presented to operate in a wide range of frequencies from 2 GHz to 9.67 GHz [32]. Furthermore, a compact band-notched wideband antenna using inverted U-shaped slots and mushroom-like EBG structures was proposed in [33], enabling dual-wideband operation including the 5.8 GHz range.

Regarding antenna array configurations, 2 × 2 patch antenna arrays were designed by the authors of [34], operated at 6 GHz, incorporating a mushroom-like EBG and triple side slotted (TSS) EBG to decrease side lobes and remove unwanted surface currents. Similarly, the EBG structures are employed in a MIMO array to minimize coupling or interference between single antenna elements [35].

These studies collectively highlight the advancements in EBG-assisted antenna designs, demonstrating significant improvements in gain, isolation, bandwidth, and SAR reduction across various applications. Continued research in this field is expected to further optimize antenna performance for emerging wireless communication technologies. In addition, the trade-off between miniaturization and performance in resonance-frequency 5.8 GHz MPAs highlights the need for innovative design techniques that achieve a compact size without sacrificing gain, bandwidth, or efficiency.

In this article, a compact, high-gain, high-efficiency MPA design is incorporated with an EBG structure for operation at the 5.8 GHz ISM band. The EBG configuration is employed to mitigate surface wave losses, enhance radiation efficiency, and enhance parameter performance, addressing critical limitations observed in conventional MPA designs. To design a high-performance compact antenna that targets the 5.8 GHz ISM band, this study investigates the optimization of electromagnetic band-gap (EBG) geometry and patch dimensions. The proposed design aims to meet the needs of WPT, RFEH, and low-power IoT sensor applications by systematically analyzing the interplay between efficiency, radiation pattern stability, and miniaturization. The antenna’s operational bandwidth, gain, directivity, and efficiency are described through simulation-based evaluations, providing insights into its potential as a candidate for integration into next-generation wireless systems that prioritize space-constrained deployments.

The paper is structured as follows: Section 2 describes the materials and methods used, including antenna modeling methodologies and EBG unit cell modeling. Additionally, it presents the characterization of EBG unit cells through reflection phase analysis and dispersion diagrams. Section 3 discusses the results, focusing on antenna parameter performance and a comparative analysis of the proposed antennas. Section 4 provides the conclusion, summarizing the key findings of the study.

## 2. Materials and Methods

### 2.1. Antenna Design Methodologies

Numerical modeling for antenna systems involves various methods and techniques to simulate and analyze performance before fabrication and integration. These methods provide valuable insights into radiation patterns, gain, voltage standing waves, impedance matching, and efficiency [36,37,38,39,40,41,42]. They help engineers to fine-tune antenna designs, optimize performance parameters, and assess system capabilities under different operating conditions.

### 2.2. Structure and Geometry of the Antenna

**Figure 2:**
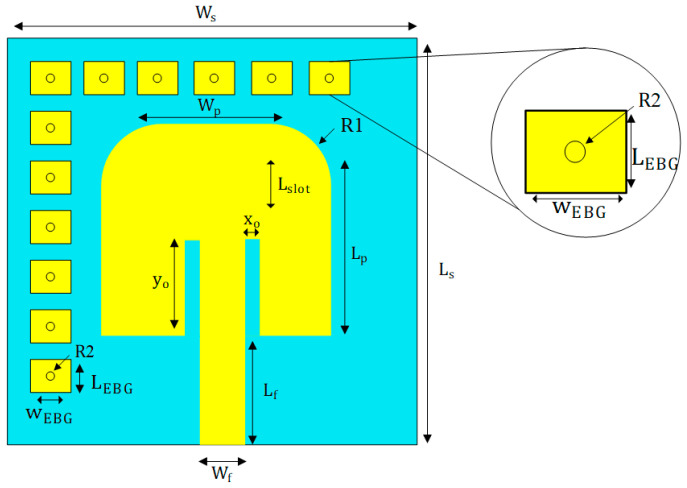
Geometrical view of EBG-based microstrip patch antenna and inset shows an EBG Unit Cell.

This study introduces a simple EBG-integrated MPA optimized for the 5.8 GHz ISM band, leveraging the Rogers 5880 substrate’s low dielectric loss (tan δ = 0.0009), with a substrate (ε_r_ = 2.2 and thickness (h_s_) = 1.6 mm) with a 0.035 mm copper patch and ground layer. The antenna design methodology combines parametric modeling in CST Microwave Studio with empirical optimization. The layout geometry of the EBG-based antenna is depicted in Figure 2. The antenna dimensions (length, width) were calculated using formulas, which were based on a given resonance frequency (f_r_) of 5.8 GHz, with the dielectric constant (ε_r_) and substrate height (h_s_) remaining constant. The key parameters are calculated as follows:

- Substrate:

The selection of substrates is a key parameter in the design, where each substrate has a specific range of thicknesses that should be considered in the antenna design and fabrication stage. Increasing the substrate thickness (h_s_) enhances the antenna’s bandwidth, though it decreases efficiency. The maximum substrate thickness is calculated using the following Equation (1):

(1) $$h_{s}\leq \frac{0.3c}{2\pi f_{r}\sqrt{\varepsilon_{r}}}$$

where c is the speed of light: 3 × 10^8^ m/s.

- Effective relative permittivity (ε_eff_):

This accounts for the combined effect of the substrate material and the surrounding air on the propagation of electromagnetic waves. It can be calculated using Equation (2):

(2) $$\varepsilon_{eff}=\frac{\varepsilon_{r}+1}{2}+\frac{\varepsilon_{r}-1}{2}{\left[1+12\frac{h_{s}}{W}\right]}^{-\frac{1}{2}}$$

W is the width of the microstrip patch.

- - Width (W*_p_*) and actual length ($L_{p}$) of the patch:

$W_{p}$ and $L_{p}$ are critical dimensions that determine its resonant frequency and radiation characteristics. These parameters are calculated using the mathematical modeling in Equations (3)–(5):

(3) $$W_{p}=\frac{c}{2f_{r}}\sqrt{\frac{\varepsilon_{r}+1}{2}}$$

(4) $$L_{p}=\frac{c}{2f_{r}\sqrt{\varepsilon_{eff}}}-2∆L,\;where\;∆L=0.412h_{s}\frac{\left(\varepsilon_{eff}+0.3\right)\left(\frac{w_{p}}{h_{s}}+0.264\right)}{\left(\varepsilon_{eff}-0.258\right)\left(\frac{w_{p}}{h_{s}}+0.8\right)}$$

(5) $$L_{eff}=L_{p}+2∆L=\frac{c}{2f_{r}\sqrt{\varepsilon_{eff}}}$$

where L_eff_ is an effective length, and ∆L is the length extension due to the field fringing effect.

The optimized dimensions of the EBG-based microstrip patch antenna design is shown in Table 1.

### 2.3. EBG Unit Cell Modeling Methodologies

**Figure 3:**
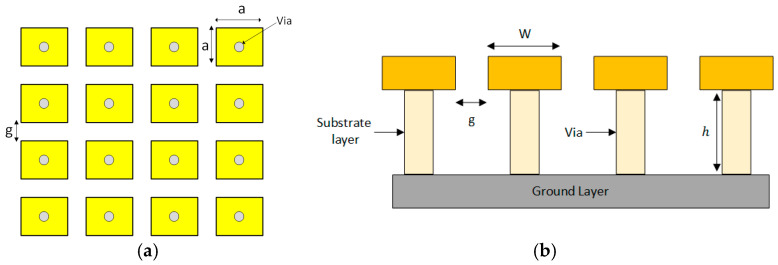
(**a**) Mushroom-like EBG structure geometry; (**b**) side view of the EBG structure.

There are many shapes of EBG structures used to suppress surface waves. Among them, the mushroom-shaped EBG structure exhibits a distinct stopband effect for surface-wave propagation. This structure consists of four main components: a ground layer, a substrate layer, a patch layer, and via, as shown in Figure 3a. The side view of the mushroom-shaped EBG structure is illustrated with its dimensions in Figure 3b.

**Figure 4:**
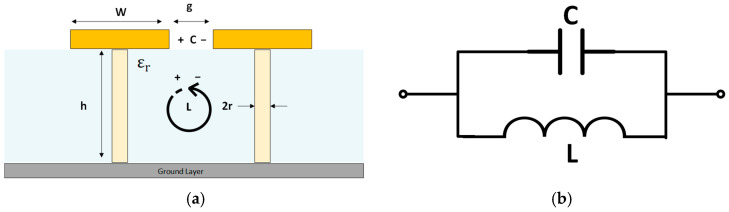
(**a**) Physical parameters of the EBG cell; (**b**) lumped element equivalent circuit of the EBG.

An LC filter array can be employed to describe the operation mechanism of the EBG structure. The gaps between adjacent patches produce the capacitive effect (C), while the inductance (L) is influenced by the current flowing through the vias. The dielectric constant (ε_r_), substrate thickness (h_s_), area (a × a), radius of via (r), and gap width (g) are the main parameters of this structure. The physical parameters of the EBG unit cell are depicted in Figure 4a, where the via has a height (h) and a radius. Figure 4b shows the lumped element equivalent circuit of the EBG, including the capacitance C and inductance L.

The formula to estimate the values of L and C for this structure is as follows in Equations (6) and (7):

(6) $$C=\frac{w\varepsilon_{0}(1+\varepsilon_{r})}{\pi }\cosh^{-1}\left(\frac{w+g}{g}\right)$$

(7) $$L=\mu h_{s},\;\mu =\mu_{0}\mu_{r}$$

where ($\varepsilon_{0}$, $\mu_{0}$) is the permittivity and permeability of the free space, respectively, and (μ_r_) is the relative permeability of the substrate Furthermore, the equivalent circuit analysis of the EBG structure demonstrates that the resulting capacitive and inductive elements possess the polarities indicated in Figure 4a.

The reflection coefficient (Γ) measures the ratio of the reflected wave to the incident wave. It is calculated using Equation (8):

(8) $$\Gamma =\frac{Z_{s}-\eta_{0}}{Z_{s}+\eta_{0}}\;;\;\left|\Gamma \right|=1,\;∠\Gamma =\pi -2\tan^{-1}\left(\frac{X}{\eta_{0}}\right)\;,\;and-90^{{}^{\circ}}\leq \Gamma \leq +90^{{}^{\circ}}$$

$Z_{s}$: Surface impedance of the EBG structure.
$\eta_{0}$: Characteristic impedance of free space (approximately 377 Ω).
$Z_{s}$: Reactance of the surface impedance ($Z_{s}=R+jX$).
∠Γ: Defines the phase of the reflected wave.

The bandwidth of the EBG structure is defined as the frequency range where the reflection phase (∠Γ) lies between +90° and −90°; this condition ensures that the surface behaves as an EBG structure, suppressing surface waves and supporting the desired electromagnetic properties. In addition, reactance (X) must lie within this range between $-\eta_{0}\;and\;+\eta_{0}$ for the EBG structure to exhibit the desired bandwidth.

The EBG surface impedance can be expressed as in Equation (9):

(9) $$Z_{s}=\frac{j\omega_{1}L}{1-{\left(\frac{\omega_{1}}{\omega_{0}}\right)}^{2}\;}\;;\;{\omega_{0}}^{2}-{\omega_{1}}^{2}=\frac{\left({\omega_{0}}^{2}L\omega_{1}\right)}{\eta_{0}}\;at\;X=\eta_{0}$$

where $\omega_{0}$ and $\omega_{1}$ are the angular frequencies.

So, from Equation (9), bandwidth can be calculated as follows in Equation (10):

(10) $$BW=\omega_{2}-\omega_{1}=\frac{{\omega_{0}}^{2}L}{\eta_{0}}=\frac{1}{120\pi }\sqrt{\frac{L}{C}}\;,\;at\;X=-\eta_{0}$$

where $\omega_{2}$ is the angular frequency

The equations from (6)–(9) are employed to determine the performance parameters of the EBG structure, including bandwidth, operational frequency, and surface impedance. These equations describe the EBG cell as having a constant gap width, denoted as ‘g’, between adjacent cells.

### 2.4. EBG Unit Cell Characterizations

The electromagnetic properties of EBG structures are often analyzed using three complementary approaches: reflection phase analysis, the suspended line technique, and dispersion diagram. Each approach offers unique insights into the behavior of the EBG structure, including its operational frequency, band-gap characteristics, and surface wave suppression ability. The following approaches regarding the mushroom-type EBG unit cell operating at 5.8 GHz are described.

#### 2.4.1. Reflection Phase Analysis

**Figure 5:**
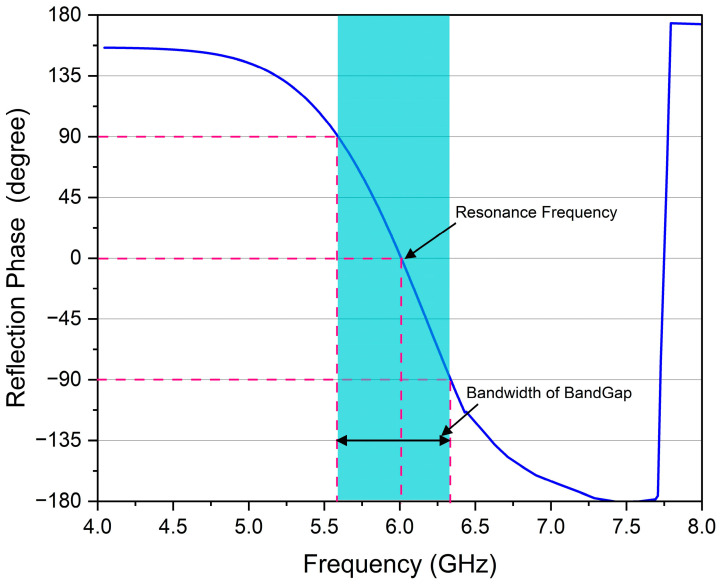
Diagram of the reflection phase characteristics for the EBG unit cell.

The typical reflection phase approach analyzes the capability of EBG to operate as an artificial magnetic conductor (AMC). It intersects 0° at the resonant frequency, enabling in-phase reflection for compact antenna integration. A sharp transition in the reflection phase from +90° to −90° occurs at a desired frequency, as shown in Figure 5, where the resonant frequency corresponds with the center frequency of the band gap identified in the dispersion diagram [43]. The high impedance characteristics in the band-gap area validate the effective performance of the EBG unit cell in suppressing surface waves. The polarization-independent characteristic of the reflection phase provides a structure suitable for applications requiring reliable performance across diverse polarization situations.

#### 2.4.2. Dispersion Diagram

**Figure 6:**
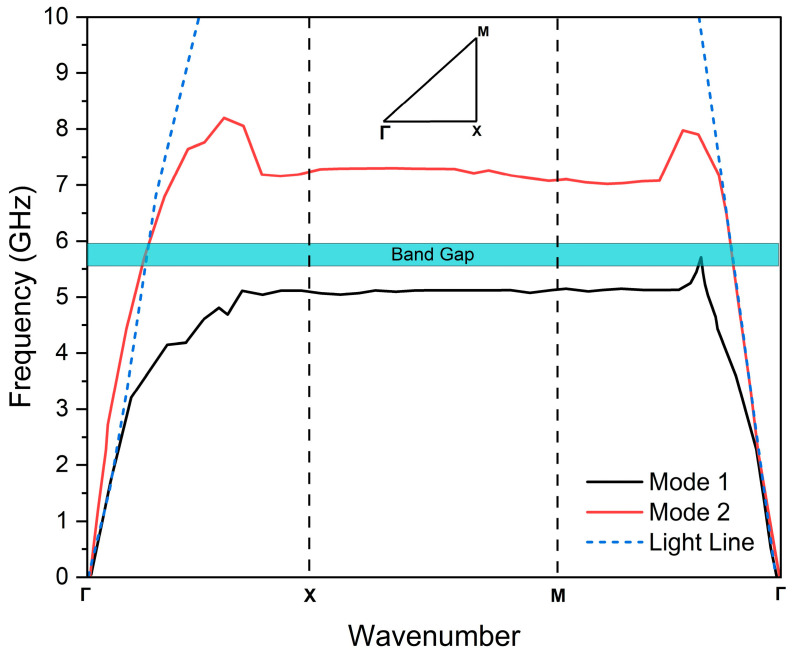
Dispersion diagram of the proposed EBG unit cell using Eigen mode analysis.

The typical dispersion diagram is a graphical method of wavenumber (k) versus frequency, utilized to determine the surface wave propagation characteristic of the EBG. The Eigen mode solver is a powerful method employed to obtain the dispersion diagram. Different modes propagate when the antenna is excited. Nevertheless, the higher modes will not propagate in the band-gap region. Figure 6 illustrates the dispersion diagram for the EBG structure at the desired frequency band [44]. Using the CST Microwave Studio, the dispersion diagram of a unit cell in the EBG structure is plotted to identify the frequency band gap. The EBG structure’s band gap was adjusted by adjusting W and g to accommodate the operational frequency.

The efficient magnetic permeability and permittivity for the designed EBG unit cell can be formulated as in Equations (11) and (12) [45]:

(11) $$\mu_{r}=\frac{2}{jk_{o}t}\;\frac{1-V_{2}}{1+V_{2}}$$

(12) $$\varepsilon_{r}=\frac{2}{jk_{o}t}\;\frac{1-V_{1}}{1+V_{1}}$$

where ($k_{o}$) indicates the wave number, (t) is the thickness of the substrate. ($V_{1}$) is the addition of S-parameters, and ($V_{2}$) represents the subtraction of S-parameters as calculated in Equations (13) and (14):

(13) $$V_{1}=S_{21}+\;S_{11}$$

(14) $$V_{2}=S_{21}-S_{11}$$

The Nicholson–Ross–Weir (NRW) technique is utilized as a conversion method to derive both permeability and permittivity from S-parameters, as presented in Equations (15)–(17) [46].

(15) $$\Gamma =X\pm \sqrt{X^{2}-1}$$

(16) $$X=\frac{S_{11}^{2}-S_{21}^{2}+1}{2S_{11}}$$

(17) $$T=\frac{S_{11}+S_{21}-\Gamma }{1-(S_{11}+S_{21})\Gamma }\;$$

where Γ represents the reflection coefficient of the circuit Γ < 1), X is the correct root, T is the transmission coefficient, $S_{11}$ is reflected signal, and $S_{21}$ is a transmitted signal.

To calculate the transmission coefficient (T) of the meta-material, Equations (18) and (19) are used:

(18) $$\ln\left(\frac{1}{T}\right)=\;\ln\left(\frac{1}{T}\right)+j(\Theta_{T}+2\pi n)$$

(19) $$n=\frac{L}{\lambda_{g}}\;$$

where n = number of roots (0, ±1, ±2), L is material length, $\lambda_{g}$ is the wavelength in a sample, and $\Theta_{T}$ is the phase of the transmission coefficient in radians.

The total count of roots can be determined by utilizing Equations (20) and (21), in addition to substituting the value obtained from Equation (21) into Equation (19).

(20) $$\frac{1}{\Lambda }=-\frac{1}{\lambda_{o}}\sqrt{\varepsilon_{r}^{o}\mu_{r}^{o}-{\left(\frac{\lambda_{o}}{\lambda_{c}}\right)}^{2}}\;$$

(21) $$\frac{1}{\Lambda }=\frac{1}{\lambda_{g}}$$

where Λ represents the complex number of wavelengths, $\varepsilon_{r}^{o}$ refers to the initial guess of material permittivity, $\mu_{r}^{o}$ is known as the initial guess of permeability, the wavelength in free space is indicated by $\lambda_{o}$, and $\lambda_{c}$ is the cut-off wavelength.

The value obtained from Equation (18) is substituted into Equation (22) as follows:

(22) $$\frac{1}{\Lambda^{2}}=-{\left[\frac{1}{2\pi L}ln\left(\frac{1}{T}\right)\right]}^{2}\;$$

The permeability ($\mu_{r}$) and permittivity ($\varepsilon_{r})$ of a material are defined in Equations (23) and (24):

(23) $$\mu_{r}=\frac{1+\Gamma }{(1+\Gamma )\Lambda \sqrt{\frac{1}{\lambda_{o}^{2}}-\frac{1}{\lambda_{c}^{2}}}}$$

(24) $$\varepsilon_{r}=\frac{\lambda_{o}^{2}}{\mu_{r}}\left[\frac{1}{\lambda_{c}^{2}}-{\left[\frac{1}{2\pi L}ln\left(\frac{1}{T}\right)\right]}^{2}\right]$$

Equation (23) defines the relative permeability ($\mu_{r}$) of a material, where Γ is the reflection coefficient at the material interface, Λ represents the propagation constant in the material, $\lambda_{o}$ is the free-space wavelength, and $\lambda_{c}$ is the cutoff wavelength of the waveguide. Equation (24) relates the relative permittivity ($\varepsilon_{r}$) to $\mu_{r}$, where $\lambda_{o}$ and $\lambda_{c}$ retain their definitions, L is the sample thickness, and T is the transmission coefficient through the material. Together, these equations describe how electromagnetic wave interactions (Γ, T) and waveguide dimensions ($\lambda_{c}$, L) determine the material’s effective $\mu_{r}$ and $\varepsilon_{r}$, which are critical for characterizing wave propagation in structured media.

## 3. Results and Discussion

This section analyzes the performance of the proposed EBG-based MPA operating at 5.8 GHz and compares it with a conventional antenna without EBG incorporation. Key parameters include the reflection coefficient (*S*_11_), gain (G), directivity (D), voltage stand wave ratio (VSWR), bandwidth (BW), radiation efficiency (η), and dimensions.

### 3.1. Reflection Coefficient (S11), Bandwidth, and VSWR

**Figure 7:**
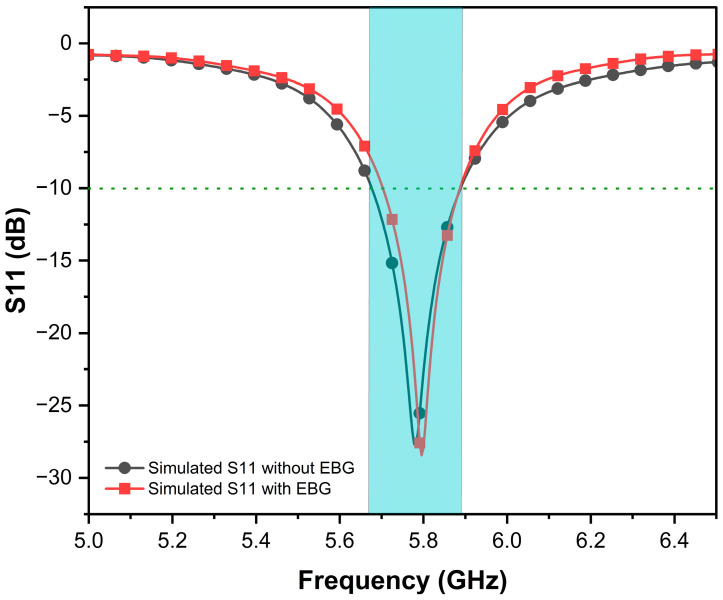
S11 versus frequency of proposed antennas.

The reflection coefficient of the proposed EBG-based antenna was simulated by CST to assess impedance matching at the resonance frequency of 5.8 GHz. The EBG-based antenna achieved a good S11 of −27.9 dB, compared to −26.4 dB for the conventional antenna design. This improvement highlights the role of the EBG structure in reducing reflections and optimizing impedance matching, as shown in Figure 7. The EBG antenna demonstrates a −10 dB bandwidth of 188 MHz as it appears in blue shadow, which is narrower than the conventional antenna’s 215 MHz bandwidth. EBG structures typically decrease antenna bandwidth due to their inherent frequency-selective behavior. However, because EBGs operate effectively only within a narrow frequency range, they inherently limit the antenna’s bandwidth. This trade-off is often acceptable in applications prioritizing efficiency, isolation, or directivity over narrow operation. In special cases, EBGs may marginally improve the bandwidth when optimized to suppress interference or enable miniaturization.

Further, the VSWR validates this enhancement by incorporating an EBG structure. The EBG antenna exhibits a good VSWR of 1.08 at resonance, confirming reduced losses due to surface wave suppression and near-perfect impedance matching, while the conventional antenna shows a slightly higher VSWR of 1.157. Both designs maintain a VSWR of less than 2 across their operational bandwidths to ensure practical usability.

### 3.2. Radiation Pattern and Efficiency

**Figure 8:**
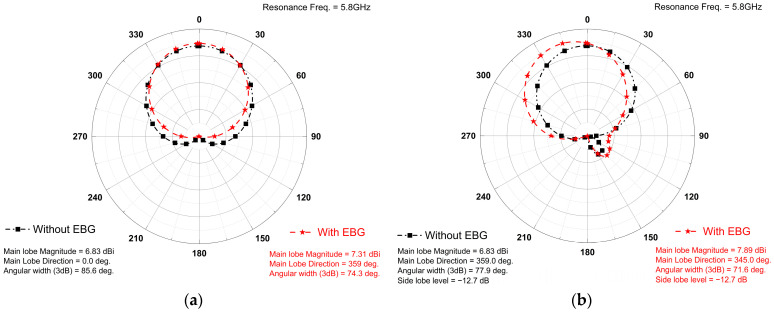
Radiation pattern of both antennas computed in (**a**) E-Plane and (**b**) H-Plane.

The radiation characteristics of the EBG-based antenna were analyzed in both the E-plane and H-plane at the resonance frequency for both antennas, as shown in Figure 8.

**Figure 9:**
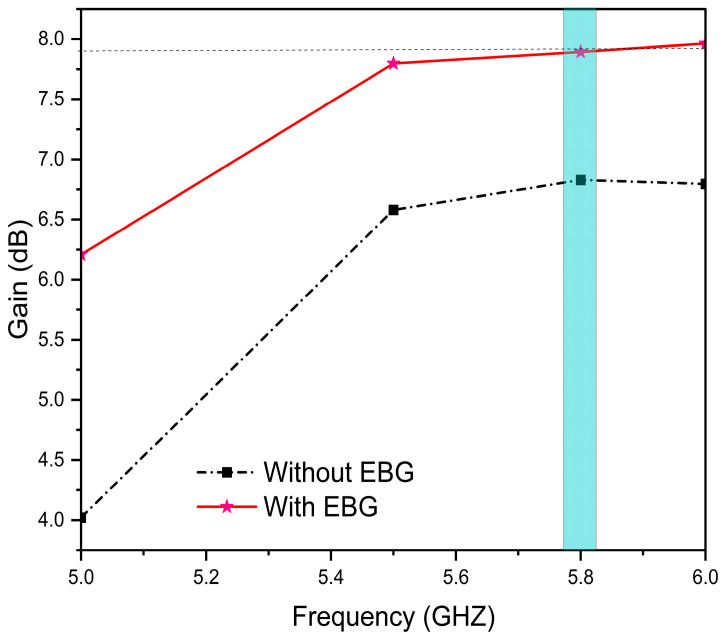
Gain (dBi) vs. frequency (GHz) of proposed designs.

The proposed EBG-based antenna shows a gain of 7.9 dBi, compared to 6.83 dBi for the conventional design, as shown in Figure 9.

**Figure 10:**
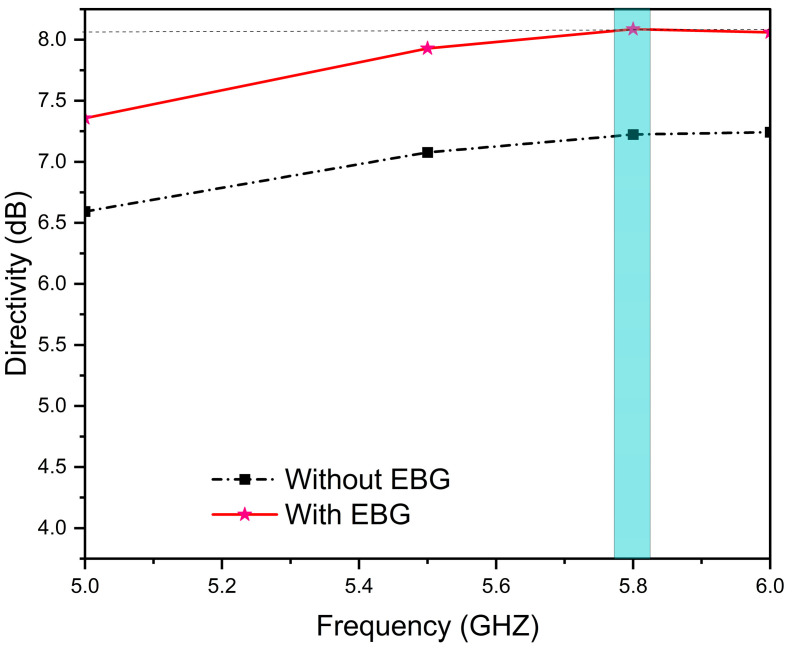
Directivity (dBi) vs. frequency (GHz) of proposed designs.

In addition, it has a higher directivity of 8.04 dBi compared to 7.24 dBi of the conventional antenna intended resonance frequency (blue shadow), as shown in Figure 10, confirming the effectiveness of the EBG structure in focusing radiated energy.

**Figure 11:**
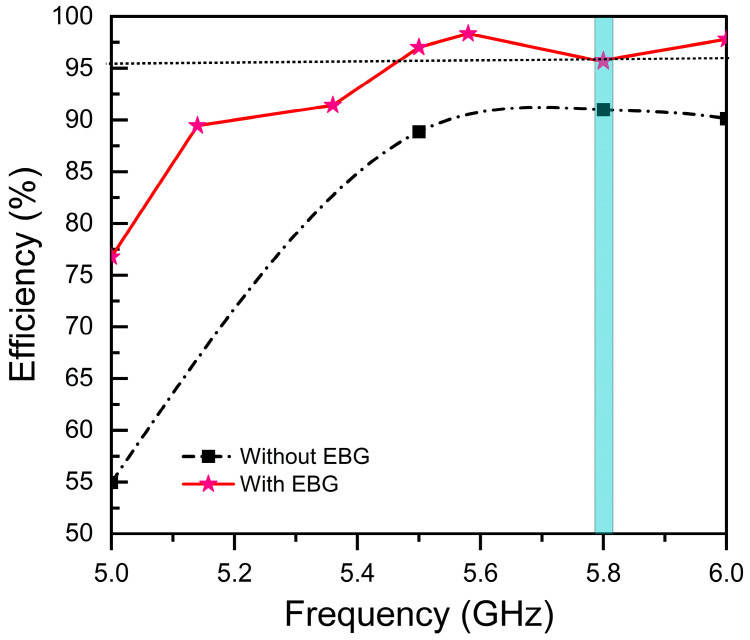
Radiation efficiency (%) vs. frequency (GHz).

However, radiation efficiency improves significantly, rising from 90.9% for a conventional design to 96.7% for an EBG-based antenna at (blue shadow) resonance frequency, as illustrated in Figure 11. The 6.3% increase in efficiency is attributed to the suppression of surface waves and substrate losses, ensuring more power is radiated rather than dissipated.

### 3.3. Z Parameter of Proposed Antennas

**Figure 12:**
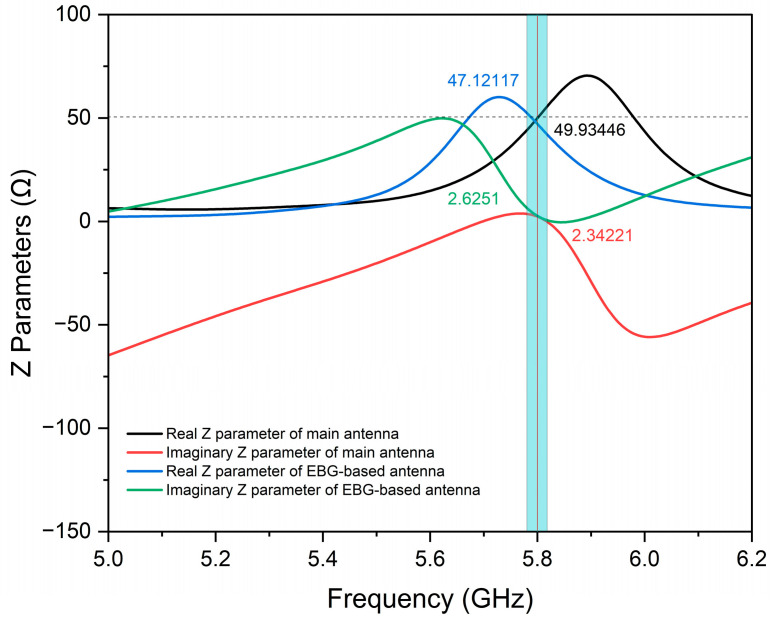
Z parameters of the proposed antennas.

Impedance matching is fundamental in radio frequency (RF) and antenna design. To achieve optimum performance, there must be a match between the antenna’s input impedance (Zin) and the port impedance, ideally 50 Ω. Simulations were conducted for each antenna design, and the input impedance (Z_in_) was extracted. The simulated value of the conventional antenna was 49.93 + j2.34 Ω, whereas the EBG-based antenna was 47.12 + j2.63 Ω at (blue shadow) resonance frequency. These values approximate the desired 50 Ω matching impedance with relatively small or zero imaginary parts, as indicated in Figure 12. The results verified that all the antenna structures are well matched to the port, facilitating efficient power delivery and low reflection losses.

### 3.4. Surface Current Distribution

**Figure 13:**
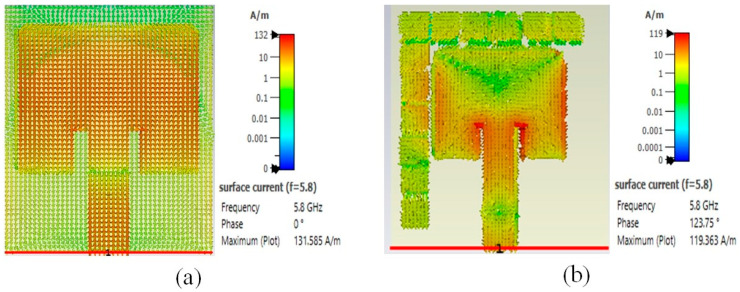
Surface current of proposed antennas: (**a**) without EBG; (**b**) with EBG.

The conventional antenna (without an EBG) exhibits a moderately high surface current of 132 A/m, where the surface current concentrates at the edges and feed points, as shown in Figure 13. On the other hand, the EBG characteristics of the EBG-based antenna show a current of 119 A/m level, which reveals that it suppresses surface waves and decreases leakage currents.

### 3.5. Experimental Results

Measuring the S-parameters of an antenna is essential for evaluating its performance, particularly in terms of impedance matching and signal transmission. The Vector Network Analyzer (VNA) is the most accurate instrument for this purpose, as it measures both the magnitude and phase of radio frequency (RF) signals. The antenna under test (AUT) must be properly mounted to minimize external interference. Additionally, a calibration kit is used to account for cable losses and connector mismatches. High-performance RF cables with SMA or N-type connectors ensure minimal signal degradation, while absorber materials help to reduce reflections in non-anechoic environments. Once calibrated, the antenna is connected to the VNA—Port 1 for reflection coefficient (S_11_) measurements or multiple ports for transmission analysis (S_21_, S_12_). The measurement environment should be controlled, ideally in an anechoic chamber, to avoid multipath interference. If testing in an open lab, RF absorbers should be placed around the antenna to minimize reflections.

The actual measurement process begins by setting the desired frequency range on the VNA, typically spanning the antenna’s operational bandwidth. For example, a Wi-Fi antenna might be tested from 2.4 GHz to 6 GHz. The number of sweep points is adjusted to balance the resolution and measurement time. The S_11_ parameter (return loss) indicates how well the antenna is matched to the transmission line, with values below −10 dB generally being considered acceptable.

**Figure 14:**
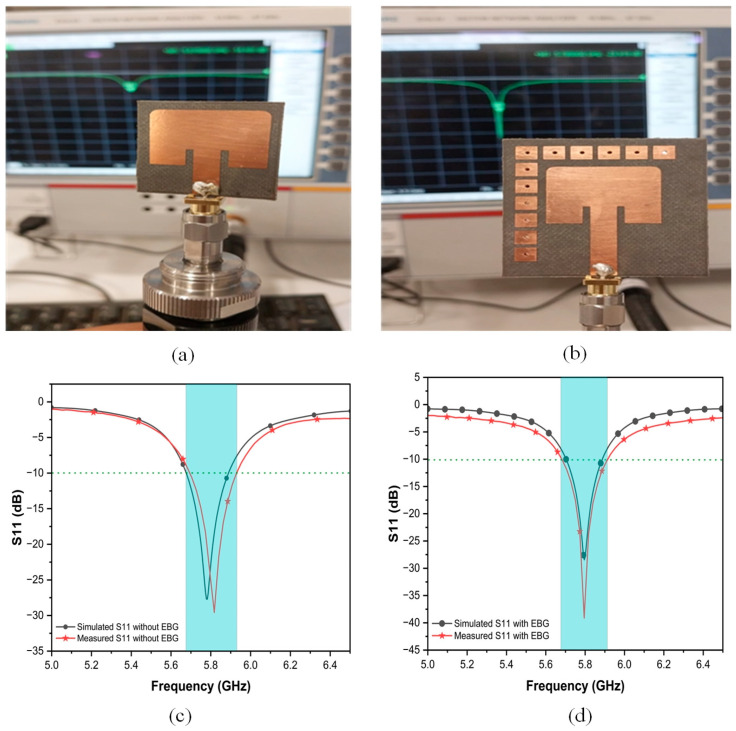
Fabricated and measured antenna prototypes (**a**) without EBG and (**b**) with EBG; measured and simulated reflection properties of antenna (**c**) without EBG and (**d**) with EBG.

Figure 14 presents a comprehensive comparison between conventional and EBG-integrated antenna prototypes through both physical implementations and electromagnetic performance analysis. The upper panels display the fabricated prototypes, with Figure 14a showing the baseline antenna design and Figure 14b demonstrating the modified version incorporating an EBG structure. The lower panels provide quantitative performance evaluation, where Figure 14c plots the reflection coefficient (S_11_) for the conventional antenna and Figure 14d shows the results for the EBG-enhanced version, each comparing simulated predictions with actual measurements. Notably, the experimental results exhibit exceptional agreement with simulations, deviating from the typical frequency shifts often observed in such comparative studies. The EBG-integrated antenna demonstrates markedly improved performance, as evidenced by a substantial reduction in the reflection coefficient magnitude. This close correlation between theoretical modeling and practical measurements not only validates the simulation approach but also confirms the effectiveness of the EBG implementation in enhancing antenna matching characteristics. The findings underscore the EBG structure’s capability to optimize antenna performance while maintaining excellent consistency between design predictions and real-world operation.

### 3.6. Energy Harvester Circuit

**Figure 15:**
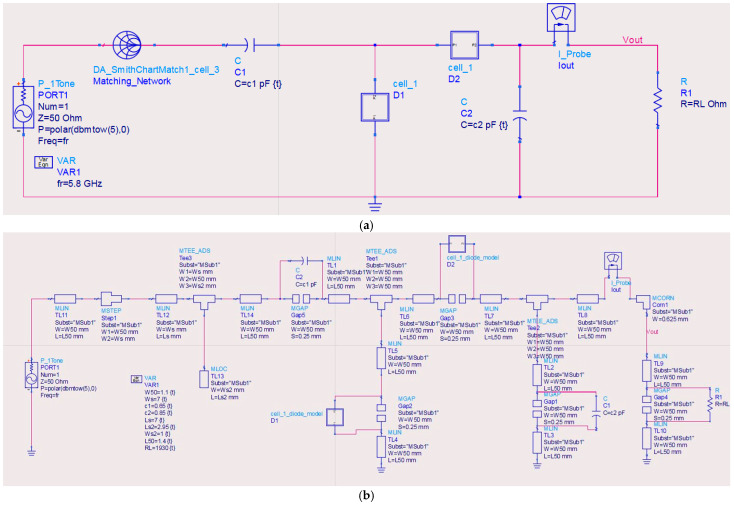

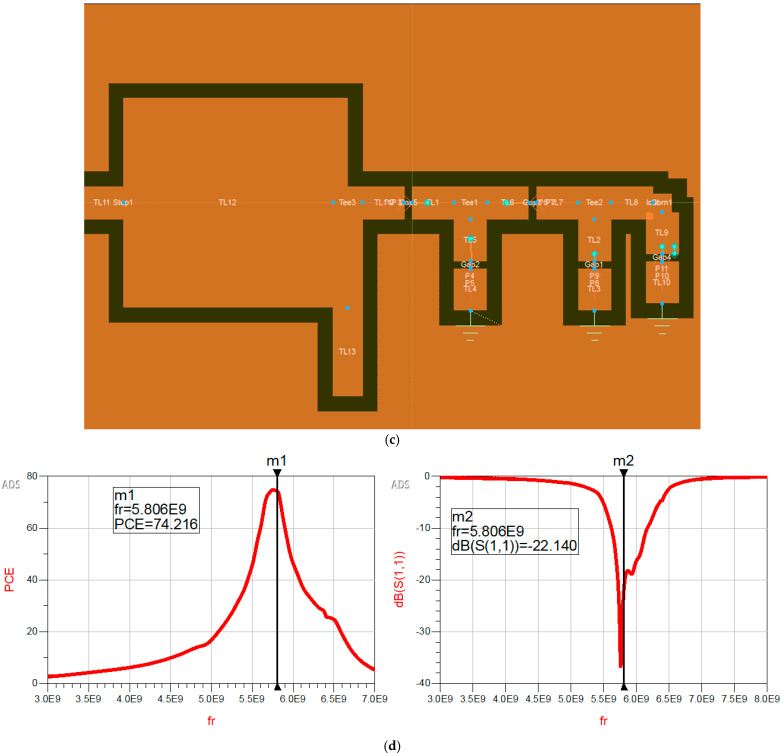
Energy harvester circuit design and performance: (**a**) schematic diagram of the circuit showing impedance matching network, two Schottky diodes (D1 and D2), and output filtering stage; (**b**) microstrip implementation with optimized microstrip line dimensions accounting for discontinuities; (**c**) final layout with parasitic considerations; and (**d**) simulated performance showing power conversion efficiency (PCE) versus frequency and input reflection coefficient (S_11_).

The proposed EBG antenna to be integrated to a RF energy harvester circuit comprises an impedance matching network, voltage multiplier rectifier, and output filtering stage, as illustrated in Figure 15. The input matching network employs a microstrip line (width (Ws) of 7.0 mm and length (Ls) of 7.0 mm) associated with an open-circuit stub (width (Ws2): 1.0 mm, length (Ls2): 2.95 mm) to transform the 50 Ω antenna impedance to the rectifier’s complex input impedance, achieving a reflection coefficient of below −22 dB. Two Schottky diodes (parasitic capacitance: 0.25 pF, parasitic inductance: 2 nH) were incorporated in the design. The output stage incorporates a simple low-pass filter that is interconnected to minimize ripple voltage across the load. Full-wave EM co-simulation in ADS Momentum accounted for layout parasitics, including substrate coupling effects and microstrip discontinuities. The optimized design demonstrated a peak power conversion efficiency (PCE) of 74.2% at 5.8 GHz for an input power of 5 dBm, with >70% efficiency maintained across a 150 MHz bandwidth, as shown in Figure 15.

### 3.7. Comparative Analysis

The performance comparison between the conventional antenna (without an EBG) and EBG-based antenna designs is presented in Table 2. The EBG antenna outperforms the conventional design in most metrics, demonstrating its suitability for high-efficiency, narrowband applications.

The most-referenced studies typically achieve matching. From Table 3, it can be seen that the EBG-based antenna demonstrated a voltage standing wave ratio (VSWR) of 1.08, which shows superior power handling. The bandwidth of 188 MHz is lower than that of some referenced values for antennas operating up to 900 MHz, but it shows strong application feasibility. Moreover, the proposed EBG-based antenna achieved a gain of 7.9 dBi, which surpassed gains reported in previous studies from 1.49 dBi to 5.21 dBi. Its efficiency is also remarkable at 95.5%, while efficiencies were noted in earlier studies, ranging from 76.5% to unspecified levels. The utilization of Rogers R5880 as the material with a reduced dielectric constant of 2.2 improves the radiation properties of the antenna over the utilization of conventional FR-4 substrates. Also, the compact size of the proposed design with dimensions of 36 × 40 × 1.57 mm^3^ properly trades size for performance. The proposed antenna yields a noteworthy improvement in performance values, making it suitable for ISM band applications and energy-harvesting systems.

## 4. Conclusions

This study demonstrated the designs and performance of a compact, high-efficiency microstrip patch antenna incorporated with a square EBG structure for RF energy harvesting in the 5.8 GHz ISM band. A miniaturized size of the proposed antenna of 36 × 40 mm^2^ (0.69λ_o_ × 0.76λ_o_) was achieved while delivering improved performance metrics: a reflection coefficient of −27.9 dB, VSWR of 1.08, gain of 7.9 dBi, directivity of 8.04 dBi, bandwidth of 188 MHz, and radiation efficiency of 95.5%. Comparison with the recent literature has confirmed its superiority over existing antennas. Integrating EBG cells effectively suppressed surface waves, enhancing the gain by 15.6%. These improvements address critical challenges in RF energy harvesting, such as minimizing losses and maximizing power transfer efficiency for battery-less IoT devices and medical applications. Notably, the compact size of the antenna and 95.5% efficiency make it a robust candidate for space-constrained, energy-sensitive wireless systems. Compared to conventional designs, the EBG-based antenna achieves improved reflection properties with a lower VSWR and enhanced bandwidth, highlighting its effective impedance-matching capabilities. The assessed design shows a strong simulation–measurement correlation without a frequency shift, and significantly lower reflection coefficients, proving its performance enhancement. This study contributes to the state-of-the-art miniaturized antenna design with improved performance, offering a viable solution for next-generation wireless technologies. Future research could explore further size reduction through metamaterial-inspired EBG geometries or experimental validation in real-world energy-harvesting scenarios. Additionally, adapting the design for multi-band operation could broaden its applicability in diverse IoT and healthcare ecosystems.

## Figures and Tables

**Table 1:** Optimized structural parameters of EBG-based antenna design.

Parameters (mm)	Dimensions (mm)	Parameters (mm)	Dimensions (mm)
Substrate length (L_s_)	40	Substrate width (W_s_)	36
Patch length ($L_{p}$)	11.8	Patch width ($W_{p}$)	10.8
Feed length (L_f_)	15	Feed width (W_f_)	4.8
Inset length (y_0_)	5	Inset width (x_0_)	1.4
EBG cell length (L_EBG_)	4	EBG cell width (W_EBG_)	4
Edge patch radius (R1)	5	Edge patch radius (R2)	0.5

**Table 2:** The performance comparison between the antenna designed with/without EBG structures. ↑ and ↓ shows an increase and decrease in the parameter of interest after embedding EBG structure in the conventional Antenna.

Parameter	Conventional Antenna (Without EBG)	EBG-Based Antenna	Trend	% Change
S11 (dB)	−22.7	−27.9	↑	22.9%
VSWR	1.157	1.08	↑	6.7%
Bandwidth (10 dB)	215MHz	188 MHz	↓	12.6%
Gain (dBi)	6.83	7.9	↑	15.6%
Directivity (dBi)	7.24	8.04	↑	11.0%
Efficiency (η)	90.9	95.5	↑	5%

**Table 3:** Performance comparison of the proposed EBG antenna with recent antennas.

Ref. (Year)	S11 (dB)	VSWR	BW (MHz)	G (dBi)	D (dBi)	Efficiency (η)	$\mathrm{Material}\;(\varepsilon_{r})$	Size (mm^3^)
[47] (2023)	−24	N/A	180	1.49	N/A	N/A	Fr-4 (4.4)	25.5 × 22.5 × 1.6
[22] (2023)	−17.49	1.31	900	5.21	N/A	N/A	Delinova 2000 (1.6)	54 × 66 × 0.5
[48] (2024)	−23.68	1.140	550	3.93	N/A	76.5%	Fr-4 (4.3)	35 × 50 × 1.6
[49] (2025)	−31.3	N/A	230	3.48	N/A	N/A	Fr-4 (4.4)	14 × 14 × 1.5
**Proposed**	−27.9	1.08	188	7.9	8.04	95.5	Roger R5880 (2.2)	36 × 40 × 1.57

## Data Availability

All data supporting the findings of this study are already included within the article.

## References

[1] Shi Q., Liu H. (2024). Energy Harvesters and Self-Powered Sensors for Smart Electronics, 2nd Edition. Micromachines.

[2] Muhammad S., Tiang J.J., Wong S.K., Smida A., Ghayoula R., Iqbal A. (2021). A dual-band ambient energy harvesting rectenna design for wireless power communications. IEEE Access.

[3] Duy P.N., Ha-Van N., Seo C. A design of 5.8 GHz rectenna array for wireless energy harvesting applications. Proceedings of the 2020 IEEE Wireless Power Transfer Conference (WPTC).

[4] Khan S., Mazhar T., Shahzad T., Bibi A., Ahmad W., Khan M.A., Saeed M.M., Hamam H. (2024). Antenna systems for IoT applications: A review. Discov. Sustain..

[5] Wang M., Fan Y., Yang L., Li Y., Feng J., Shi Y. (2019). Compact dual-band rectenna for RF energy harvest based on a tree-like antenna. IET Microw. Antennas Propag..

[6] Adu-Manu K.S., Adam N., Tapparello C., Ayatollahi H., Heinzelman W. (2018). Energy-harvesting wireless sensor networks (EH-WSNs) A review. ACM Trans. Sens. Netw..

[7] Salama R., Al-Turjman F. (2023). Sustainable Energy Production in Smart Cities. Sustainability.

[8] Albaihani Y., Akram R., Almohaimeed Z., Almohaimeed A., Hajlaoui E.A. (2025). Optimal antenna design for wireless energy harvesting system in ISM band. Results Phys..

[9] Taqdeer M.M., Amjad Q.M., Zahid M., Amin Y. 2 × 2 Hexagonal Shaped Antenna Array for 5.8 GHz ISM Band Applications. Proceedings of the 7th International Multi-Topic ICT Conference.

[10] Aliqab K., Lavadiya S., Alsharari M., Armghan A., Daher M.G., Patel S.K. (2023). Design and Fabrication of a Low-Cost, Multiband and High Gain Square Tooth-Enabled Metamaterial Superstrate Microstrip Patch Antenna. Micromachines.

[11] Tangel C., Teşneli N.B. (2024). Teşneli, and Applications, A wideband, thin profile and enhanced gain microstrip patch antenna modified by novel mushroom-like EBG and periodic defected ground structures. J. Electromagn. Waves Appl..

[12] Yang Y., Wang H., Jiang R., Guo X., Cheng J., Chen Y. (2022). A Review of IoT-Enabled Mobile Healthcare: Technologies, Challenges, and Future Trends. IEEE Internet Things J..

[13] Anitha V.R., Palanisamy S., Khalaf O.I., Algburi S., Hamam H. (2024). Design and analysis of SRR based metamaterial loaded circular patch multiband antenna for satellite applications. ICT Express.

[14] Patel D.M., Raval F. (2024). Gain improvement of HMSIW Antenna with SRRs. E-Prime-Adv. Electr. Eng. Electron. Energy.

[15] Arora C. Metamaterial Based Multiband Microstrip Patch Antenna with Defective Ground. Proceedings of the 2024 International Conference on Communication, Control, and Intelligent Systems (CCIS).

[16] Nurhayati N., Zulkifli F.Y., Setijadi E., Sukoco B.E., Yasin M.N.M., De Oliveira A.M. (2024). Bandwidth, gain improvement, and notched-band frequency of SWB Wave Coplanar Vivaldi antenna using CSRR. IEEE Access.

[17] Olan-Nuñez K.N., Murphy-Arteaga R.S. (2023). Murphy-Arteaga, Dual-Band Antenna on 3D-Printed Substrate for 2.4/5.8 GHz ISM-Band Applications. Electronics.

[18] Pande S.V., Patil D.P. (2024). Miniaturization of antenna using metamaterial loaded with CSRR for wireless applications. Bull. Electr. Eng. Inform..

[19] Akgol O., Altintas O., Unal E., Karaaslan M., Karadag F. (2018). Linear to left-and right-hand circular polarization conversion by using a metasurface structure. Int. J. Microw. Wirel. Technol..

[20] Ramli N.H., Lokman M.A.F., Bahari N., Zahid L., Yob R.C., Mat M.H., Jamlos M.A., Alzubaidi L.H., Hussein A.H.A. Left-Handed Metamaterial Wearable Antenna at 5G Frequency Range for Wireless Body Area Network. Proceedings of the 2023 3rd International Conference on Mobile Networks and Wireless Communications (ICMNWC).

[21] Rana S., Jain P. (2024). Utilizing characteristics mode analysis for designing a miniaturised high-gain metasurface antenna with wideband circular polarization and reduced scattering. J. Electromagn. Waves Appl..

[22] Ash-Shiddiq H.A., Ryanu H.H., Nur L.O. (2023). Wearable Antenna Dual Band With Electromagnetic Band Gap (Ebg) Structure For Health Applications. J. Comput. Eng. Prog. Appl. Technol..

[23] Almohaimeed A.M., Hajlaoui E.A., Almohaimeed Z.M. (2023). Single Band EBG Antenna for Wireless Power Transfer Applications. Comput. Syst. Sci. Eng..

[24] Jain S., Purohit G., Ghosh S., Saha C. Design and Analysis of 4x4 EBG Backed Miniaturized Disc-shaped Wearable RFID Tag Antenna for Biomedical Communications. Proceedings of the 2024 IEEE Space, Aerospace and Defence Conference (SPACE).

[25] Amalraj T., Savarimuthu R. (2019). Design and Analysis of Microstrip Patch Antenna Using Periodic EBG Structure for C-Band Applications. Wirel. Pers. Commun..

[26] Sultana S., Basak R. (2019). Performance Evaluation of Meander Line Implantable Antenna integrated with EBG Based Ground for Anatomical Realistic Model. AIUB J. Sci. Eng. (AJSE).

[27] Laskar M.I., Basu B., Nandi A. (2024). A multiband slot antenna with groundless EBG structure for wearable WLAN/WiMAX applications. Int. J. Electron..

[28] Khemchandra A., Pinku R. Electromagnetic Band Gap-Based Circular Ring-Shaped Wearable Antenna With Improved Gain For Internet of Things Applications in 5-G Sub-6 GHz. Proceedings of the 2024 ITU Kaleidoscope: Innovation and Digital Transformation for a Sustainable World (ITU K).

[29] Sufian M.A., Abbas A., Choi D., Lee J., Hussain N., Kim N. Quad-Port MIMO Antenna Design with Low SAR for 3.5 GHz 5G and 5.8 GHz ISM Bands. Proceedings of the 2024 IEEE Joint International Symposium on Electromagnetic Compatibility, Signal & Power Integrity: EMC Japan/Asia-Pacific International Symposium on Electromagnetic Compatibility (EMC Japan/APEMC Okinawa).

[30] Alnaiemy Y., Nagy L. Further Investigation of The Feasibility of Using EBG Structure-Based Microstrip Antenna for Gain Enhancement. Proceedings of the 2020 International Conference on Radar, Antenna, Microwave, Electronics, and Telecommunications (ICRAMET).

[31] Keshwani V.R., Bhavarthe P.P., Rathod S.S. (2022). Compact Embedded Dual Band EBG Structure with Low SAR for Wearable Antenna Application. Prog. Electromagn. Res. M.

[32] Goswami P.K. (2018). A Reconfigurable Compact Size Fractal Antenna for UWB RF Energy Harvesting. Int. J. Microw. Opt. Technol..

[33] Abdalla M.A., Al-Mohamadi A.A., Mohamed I.S. (2019). A miniaturized dual band EBG unit cell for UWB antennas with high selective notching. Int. J. Microw. Wirel. Technol..

[34] Abdulhameed M.K., Isa M.S.B.M., Zakaria Z., Ibrahim I.M., Mohsen M.K., Attiah M.L., Dinar A.M. (2019). Enhanced performance of compact 2 × 2 antenna array with electromagnetic band-gap. Microw. Opt. Technol. Lett..

[35] Elabd R.H., Al-Gburi A.J. (2024). Super-compact 28/38 GHz 4-port MIMO antenna using metamaterial-inspired EBG structure with SAR analysis for 5G cellular devices. J. Infrared Millim. Terahertz Waves.

[36] Gibson W.C. (2021). The Method of Moments in Electromagnetics.

[37] Hasan A.A., Kvasnikov A.A., Klyukin D.V., Ivanov A.A., Demakov A.V., Mochalov D.M., Kuksenko S.P. (2023). On modeling antennas using mom-based algorithms: Wire-grid versus surface triangulation. Algorithms.

[38] Szabó B., Babuška I. (2021). Finite Element Analysis: Method, Verification and Validation.

[39] David D.S.K., Jeong Y., Wu Y.C., Ham S. (2023). An Analytical Antenna Modeling of Electromagnetic Wave Propagation in Inhomogeneous Media Using FDTD: A Comprehensive Study. Sensors.

[40] Alquaydheb I.N., Alfawaz S., Avval A.G., Ghayouraneh S., El-Ghazaly S. Choke ring circular waveguide antenna analysis and design using geometrical theory of diffraction. Proceedings of the 2022 IEEE International Symposium on Antennas and Propagation and USNC-URSI Radio Science Meeting (AP-S/URSI).

[41] Shariff B.G.P., Pathan S., Mane P.R., Ali T. (2024). Characteristic mode analysis based highly flexible antenna for millimeter wave wireless applications. J. Infrared Millim. Terahertz Waves.

[42] Xia Z., Liu J., Li Z., Song J. A Fast Computing Technology of Three-dimensional Multilevel Fast Multipole Algorithm Based on Deep Learning. Proceedings of the 2024 Photonics & Electromagnetics Research Symposium (PIERS).

[43] Ashyap A.Y.I., Bin Dahlan S.H., Abidin Z.Z., Abbasi M.I., Kamarudin M.R., Majid H.A., Dahri M.H., Jamaluddin M.H., Alomainy A. (2020). An Overview of Electromagnetic Band-Gap Integrated Wearable Antennas. IEEE Access.

[44] Mohamadzade B., Afsahi M. (2017). Mutual Coupling Reduction and Gain Enhancement in Patch Array Antenna Using a Planar Compact Electromagnetic Band Gap (EBG) Structures. IET Microw. Antennas Propag..

[45] Hossain M.B., Faruque M.R., Islam S.S., Islam M.T. (2021). Modified double dumbbell-shaped split-ring resonator-based negative permittivity metamaterial for satellite communications with high effective medium ratio. Sci. Rep..

[46] Iqbal K., Khan Q.U. (2022). Review of Metasurfaces Through Unit Cell Design and Numerical Extraction of Parameters and Their Applications in Antennas. IEEE Access.

[47] Atif A., Majid A., Zahid M., Amin Y. (2023). Circular Slotted Triangular Patch Antenna for 5.8 GHz ISM Band Applications. Eng. Proc..

[48] Guneser M.T., Seker C., Guler M.I., Fitriyani N.L., Syafrudin M. (2024). Efficient 5.8 GHz Microstrip Antennas for Intelligent Transportation Systems: Design, Fabrication, and Performance Analysis. Mathematics.

[49] Raut H., Kingsly S., Subbaraj S., Malekar R. (2025). EBG-Based Low Profile Corrugated Antenna for 5G Applications in Sub-6 GHz Spectrum. Prog. Electromagn. Res. Lett..

